# Serum Bile Acids Improve Prediction of Alzheimer's Progression in a Sex‐Dependent Manner

**DOI:** 10.1002/advs.202306576

**Published:** 2023-12-13

**Authors:** Tianlu Chen, Lu Wang, Guoxiang Xie, Bruce S. Kristal, Xiaojiao Zheng, Tao Sun, Matthias Arnold, Gregory Louie, Mengci Li, Lirong Wu, Siamak Mahmoudiandehkordi, Matthew J. Sniatynski, Kamil Borkowski, Qihao Guo, Junliang Kuang, Jieyi Wang, Kwangsik Nho, Zhenxing Ren, Alexandra Kueider‐Paisley, Colette Blach, Rima Kaddurah‐Daouk, Wei Jia, Alzheimer's Disease Neuroimaging Initiative (ADNI) and the Alzheimer Disease Metabolomics Consortium (ADMC)

**Affiliations:** ^1^ Center for Translational Medicine Shanghai Sixth People's Hospital Affiliated to Shanghai Jiao Tong University School of Medicine Shanghai 200233 China; ^2^ School of Chinese Medicine Hong Kong Baptist University Kowloon Tong Hong Kong 999077 China; ^3^ Human Metabolomics Institute Shenzhen 518109 China; ^4^ Division of Sleep and Circadian Disorders Department of Medicine Brigham and Women's Hospital Boston MA 02115 USA; ^5^ Division of Sleep Medicine Harvard Medical School Boston MA 02115 USA; ^6^ Department of Psychiatry and Behavioral Sciences Duke University Durham NC 27710 USA; ^7^ Institute of Bioinformatics and Systems Biology Helmholtz Zentrum München German Research Center for Environmental Health 85764 Neuherberg Germany; ^8^ West Coast Metabolomics Center Genome Center University of California Davis Davis CA 95616 USA; ^9^ Department of Radiology and Imaging Sciences and the Indiana Alzheimer Disease Center Indiana University School of Medicine Indianapolis IN 46202 USA; ^10^ Duke Molecular Physiology Institute Duke University Durham NC 27708 USA; ^11^ Duke Institute of Brain Sciences Duke University Durham NC 27708 USA; ^12^ Department of Medicine Duke University Durham NC 27708 USA

**Keywords:** alzheimer's disease, bile acid, cholesterol, mild cognitive impairment, sex difference

## Abstract

Sex disparities in serum bile acid (BA) levels and Alzheimer's disease (AD) prevalence have been established. However, the precise link between changes in serum BAs and AD development remains elusive. Here, authors quantitatively determined 33 serum BAs and 58 BA features in 4 219 samples collected from 1 180 participants from the Alzheimer's Disease Neuroimaging Initiative. The findings revealed that these BA features exhibited significant correlations with clinical stages, encompassing cognitively normal (CN), early and late mild cognitive impairment, and AD, as well as cognitive performance. Importantly, these associations are more pronounced in men than women. Among participants with progressive disease stages (n = 660), BAs underwent early changes in men, occurring before AD. By incorporating BA features into diagnostic and predictive models, positive enhancements are achieved for all models. The area under the receiver operating characteristic curve improved from 0.78 to 0.91 for men and from 0.76 to 0.83 for women for the differentiation of CN and AD. Additionally, the key findings are validated in a subset of participants (n = 578) with cerebrospinal fluid amyloid‐beta and tau levels. These findings underscore the role of BAs in AD progression, offering potential improvements in the accuracy of AD prediction.

## Graphical abstract

There are sex differences in serum bile acid (BA) levels and Alzheimer's disease (AD) incidence. With a longitudinal ADNI study, it is found that BAs trend in relation to AD progression and the alteration is earlier in men than women. Sex‐specific features associated with disease stages, cognitive functions, and progression highlighted BA roles in AD progression.

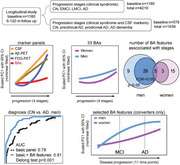

## 1 Introduction

Alzheimer's disease (AD) affects millions of people and puts a growing strain on healthcare systems around the world. To date, there have been no effective therapies for preventing or slowing the progression of AD. Epidemiological studies have confirmed differences in the risk and severity of AD between men and women, but the underlying mechanisms are only beginning to be understood.^[^ ^1^ ^]^ Metabolomics, the study of small molecules in living organisms, has the potential to identify biomarkers and understand the development of mild cognitive impairment (MCI) and AD.^[^ ^2^ ^]^ In our previous studies,^[^ ^2^, ^3^ ^]^ we used metabolomics to identify metabolic signatures related to disease and disease progression in serum, cerebrospinal fluid (CSF), and brain samples from the Alzheimer's Disease Neuroimaging Initiative (ADNI) and Religious Orders Study and Rush Memory and Aging (ROS‐MAP) cohorts. We also examined sex effect on the relationship between metabolic changes and AD, which may help explain the observed differences in AD susceptibility and severity between men and women.^[^ ^3h^ ^]^

Bile acids (BAs), a group of metabolites involved in cholesterol catabolism, have received significant attention due to their important biological characteristics and functions.^[^ ^4^ ^]^ Primary BAs (CA and CDCA) are synthesized in hepatocytes from cholesterol, while secondary BAs are biotransformed by gut bacteria from primary BAs.^[^ ^2a^ ^]^ As a result, changes in BA profiles may reflect changes in cholesterol metabolism and bacterial functions, both of which have been linked to AD. Previous research has shown that BA profiles are significantly altered in patients with mild cognitive impairment (MCI) and AD compared to cognitively normal (CN) individuals.^[^ ^5^ ^]^ Higher levels of secondary BAs and their conjugated forms and higher ratios of secondary to primary BAs are associated with worse amyloid‐beta, tau, and neuropil markers and cognitive function.^[^ ^5a,b^ ^]^ Lower levels of primary BAs have been linked to increased brain amyloid deposits, faster accumulation of white matter lesions, and increased brain atrophy.^[^ ^6^ ^]^ We have previously suggested that changes in BA profiles may play a role in the development of AD and that the gut microbiome‐bile acid‐brain cholesterol axis may be a potential target for the prevention and treatment of AD.^[^ ^5^, ^7^ ^]^ In addition, we and others have observed that BA profiles are affected by sex, and sex differences should be considered when studying BA changes related to diseases.^[^ ^8^ ^]^

**Figure 1:**
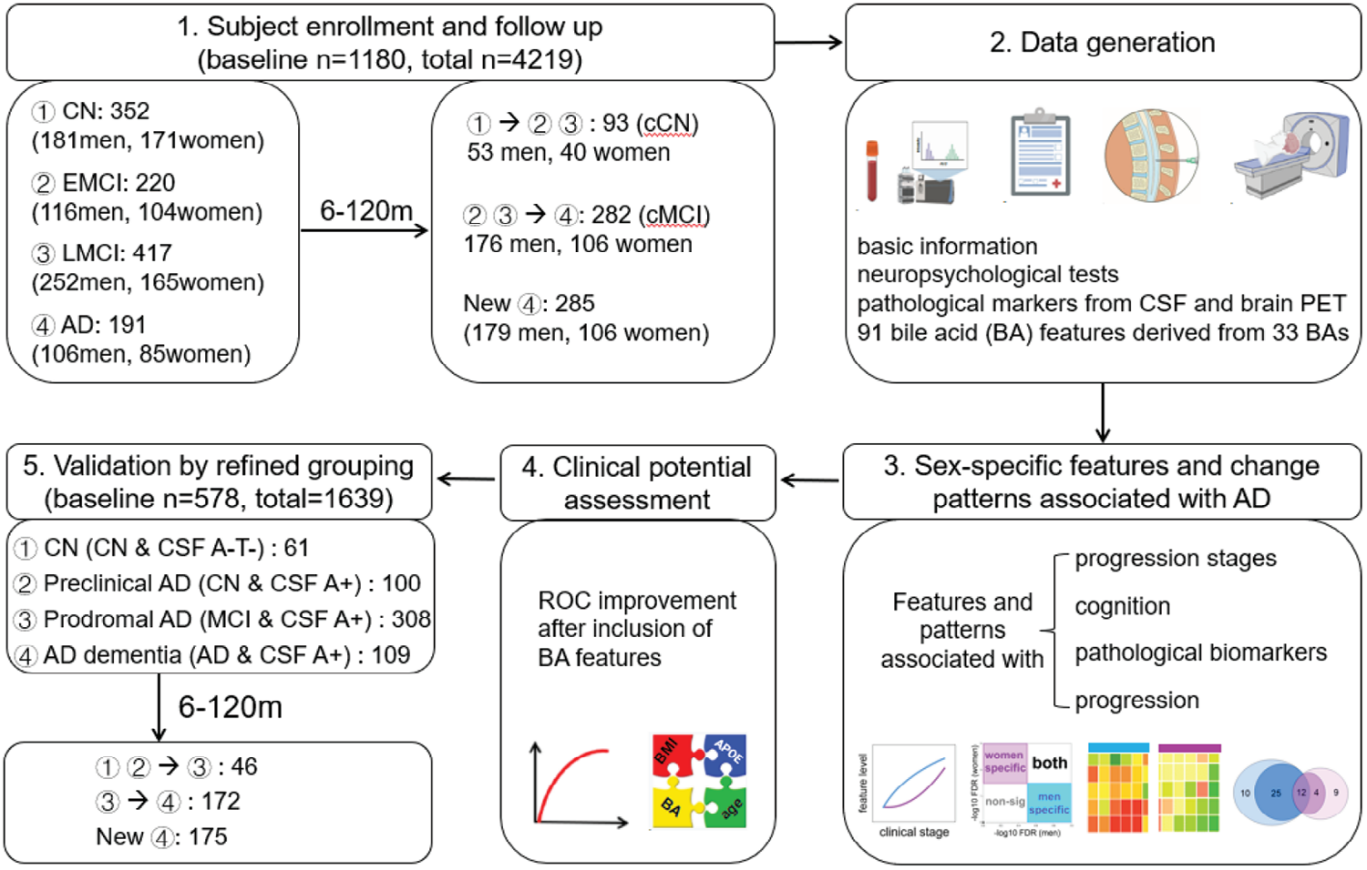
Study pipeline. CN = Cognitively normal, MCI = mild cognitive impairment, EMCI = Early mild cognitive impairment, LMCI = Late mild cognitive impairment, AD = Alzheimer's disease, cCN = converted CN, cMCI = converted MCI, CSF = cerebrospinal fluid, PET = Positron Emission Tomography, A = amyloid‐beta, T = tau.

Here, we examined the concentration of 33 BAs in ADNI samples from multiple centres. Besides their concentrations, we also analysed the percentages of individual BAs relative to the total concentration of BAs (TBA) and calculated ratios that reflect enzymatic activities and gut microbiota functions. We depicted progression patterns of BA profiles and identified feature panels specific to men and women. We demonstrated the enhancement ability of BA features to clinical biomarkers for disease stage differentiation and prediction (**Figure** **1**). Findings were validated in a sub set of subjects with CSF amyloid‐beta (A) and tau (T) measurements. Our goal is to replicate and refine previous findings, test hypotheses, and increase our understanding of the gut microbiome‐bile acid‐brain cholesterol axis in AD.

## 2 Results

### 2.1 Study Samples and Bile Acid Features

A total of 1180 subjects (baseline) participated in this study, 655 men and 525 women (**Table** **1**). Among them, 352, 220, 417, and 191 subjects were clinically diagnosed with CN, early MCI (EMCI), late MCI (LMCI), and AD respectively. During 6–120 months follow‐up (mean duration was 33.2 months), 93 CNs (53 men and 40 women) progressed to MCI (named cCN, Table S1, Supporting Information), 282 MCIs (176 men and 106 women) progressed to AD (named cMCI, Table S2, Supporting Information), and 285 were newly diagnosed as AD (179 men and 106 women; 282 were progressed from MCI and 3 were progressed from CN). A total of 4219 (2386 men and 1833 women) serum samples (94% fasting) collected at baseline and follow‐up visits were involved in this study (Table S3, Supporting Information). A total of 33 BAs were quantitatively measured and 58 extended features were generated including percent concentrations of each BA to TBA and BA ratios reflective of enzymatic activities and gut microbiota functions (Table S4, Supporting Information). Pathological markers were categorized into 3 panels according to how they were obtained, namely, CSF, Aβ‐PET, and FDG‐PET.

**Table 1:** Demographic and clinical characteristics of the 1180 baseline samples.

	All [n = 1180]		CN [n = 352]		EMCI [n = 220]		LMCI n = 417]		AD n = 191]	
	men	women	men	women	men	women	men	women	men	women
N	655	525	181	171	116	104	252	165	106	85
Age [yr]	73.94 ± 6.87	72.63 ± 7.24	74.92 ± 6.06	73.68 ± 5.33	71.03 ± 6.81	69.95 ± 7.96	74.33 ± 6.95	72.39 ± 7.65	74.57 ± 7.3	74.29 ± 7.97
BMI [kg m^−2^]^b)^	26.94 ± 3.79	26.48 ± 5.29	26.91 ± 3.58	26.86 ± 5.12	28.01 ± 4.61	28.17 ± 6.25	26.68 ± 3.47	25.89 ± 5.18	26.41 ± 3.67	24.82 ± 3.71
Education [yr]^a)b)^	16.45 ± 2.78	15.31 ± 2.8	17.1 ± 2.55	15.6 ± 2.71	16.55 ± 2.59	15.5 ± 2.72	16.29 ± 2.83	15.5 ± 2.79	15.62 ± 2.96	14.11 ± 2.82
*APOE*(e4+) [%]^a)b)^	48%	45%	27%	30%	46%	38%	54%	55%	72%	64%
MMSE^a)b)^	27.25 ± 2.55	27.45 ± 2.5	29.03 ± 1.14	29.14 ± 1.07	28.29 ± 1.46	28.47 ± 1.64	27.19 ± 1.9	27.11 ± 1.8	23.24 ± 2.08	23.46 ± 1.85
ADAS‐11^a)b)^	10.9 ± 6.02	9.6 ± 6.12	6.46 ± 2.86	5.19 ± 2.74	8.22 ± 3.39	7.64 ± 3.51	11.83 ± 4.3	11.11 ± 4.84	19.16 ± 6.49	17.93 ± 6.23
ADAS‐13^a)b)^	17.03 ± 8.63	15.33 ± 9.35	10.11 ± 4.24	8 ± 3.91	12.91 ± 5.04	11.92 ± 5.33	18.9 ± 6.02	18.32 ± 7.25	29.34 ± 7.78	28.49 ± 7.71
FDG‐PET [%]^a)b)^	73%	68%	74%	64%	99%	99%	62%	64%	70%	49%
Aβ‐PET [%]^a)b)^	42%	46%	43%	46%	100%	99%	20%	30%	27%	14%
CSF [%]^a)b)^	68%	70%	65%	68%	91%	92%	62%	62%	64%	59%

^a),b)^ represent Chi‐squared test or analysis of variance p<0.05 among 4 groups in men and women respectively. CN = cognitively normal, EMCI = early mild cognitive impairment, LMCI = late mild cognitive impairment, AD = Alzheimer's disease, *APOE* = apolipoprotein E ε4 genotype, MMSE = minimal mental state examination total score, ADAS = Alzheimer's disease assessment scale‐cognitive subscale score, CSF = cerebrospinal fluid, Aβ‐PET = Aβ positron emission tomography, FDG‐PET = 18F‐fluorodeoxyglucose positron emission tomography.

### 2.2 Serum BA Profiles were Associated with Clinical Stages in a Sex‐Dependent Manner

**Figure 2:**
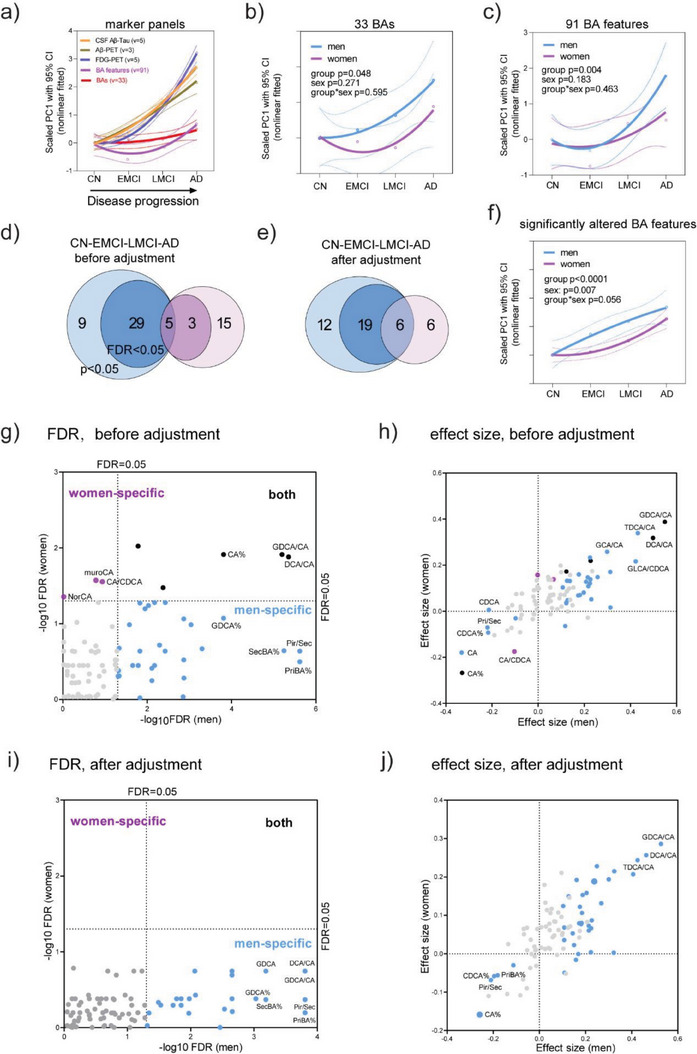
BA profiles were associated with clinical stages in a sex‐dependent manner. a) Trajectory (nonlinear fitted curves with 95% CI of PC1, the first component of PCA) of different marker panels across clinical stages (CN, EMCI, LMCI, and AD). PCA trajectories of 33 BAs b) and 91 BA features c) in men (blue) and women (purple). The p values were from two‐way ANOVA. The numbers of significantly altered BA features in men and women identified by mixed linear models before d) and after e) adjustment of covariates (age, *APOE‐4*, education year, BMI, medications, cohort, and fasting status). f) Trajectory of significantly (mixed linear regression model, no adjustment of covariates, FDR<0.05) altered BA features (34 for men and 8 for women) in men (blue) and women (purple) across 4 clinical stages. The FDR and effect size (β) values of sex‐specific (men in blue and women in purple) and common (black) features associated with clinical stages, which were identified by mixed linear regression models before g,h) and after i,j) the adjustment of covariates (age, *APOE‐4*, education year, BMI, medications, cohort, and fasting status). Features were z‐score scaled before being fed to the models and thus the effect sizes were comparable.

Principal component analysis (PCA) was conducted on BAs, BA features, and pathological marker panels respectively and the first component, PC1, was taken as the representative variable. Compared with pathological marker panels, the alterations of BAs (red curve in **Figure** **2**a) were less apparent than those of CSF and PET panels. Changes of 33 BAs were earlier and faster in men than in women (Figure 2b). The change pattern (Figure 2a,c) of 91 BA features (33 BAs and 58 extended features) were not completely consistent to that of 33 BAs, probably due to the inclusion of noise features. These observations motivated us to identify sex‐specific feature panels associated with clinical stages.

Mixed linear models were built and BA features that were significantly altered in 4 clinical stages were identified, in men and women separately. There were more altered features in men than in women, regardless of whether the covariates (age at sampling, *APOE‐4*, education year, BMI, medications, cohort, and fasting status) were adjusted or not and regardless of the significance levels (Figure 2d,e). There was no significantly altered feature in women when covariates were adjusted and FDR<0.05 was taken as the significance level. The trajectories based on sex‐specific feature panels (before adjustment, FDR<0.05; 34 for men and 8 for women) signified that for men, BAs changed early and fast from CN and then the changes slowed down gradually in MCI and AD, while for women, the apparent change came later until EMCI and then changes were fast in subsequent groups (Figure 2f). Figure 2g–j showed the p and effect size values of sex‐specific and common features associated with clinical stages. Before the adjustment of covariates, lower CA% and higher GDCA/CA and DCA/CA were associated with disease severity in both men and women. Lower Pri/Sec in men and lower CA/CDCA in women were associated with disease severity. After the adjustment of covariates, lower PriBA% and Pri/Sec and higher GDCA/CA and DCA/CA were associated with disease severity in men. Together, changes in serum BA profiles were earlier and more dramatic in men than women.

### 2.3 BA features were Associated with Clinical Markers in a Sex‐Dependent Manner

**Figure 3:**
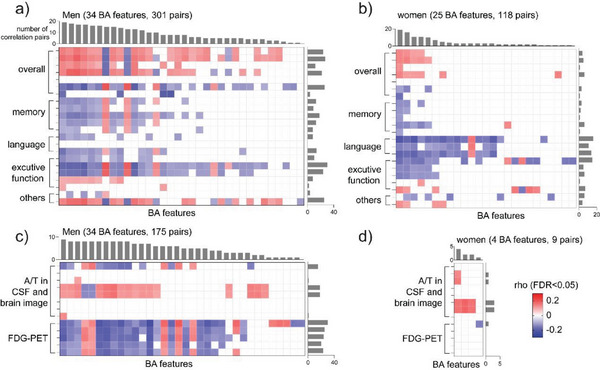
BA profiles were associated with clinical markers in a sex‐dependent manner. Heatmaps of the correlation coefficients between 91 BA features and clinical markers from neuropsychological tests a,b) and CSF and PET imaging c,d) in men and women. Blank, non‐significant (FDR>0.05); pink, positive association; blue, negative association. The adjusted covariates include age at sampling, *APOE‐4*, education year, BMI, medications, cohort, and fasting status.

Consistent with previous findings, correlation analysis of BA features and clinical markers showed that more correlated pairs were detected in men than women regardless of whether the covariates were adjusted or not. There were 34 and 25 BA features correlated with cognition and 34 and 4 BA features correlated with A/T markers in men and women respectively, after the adjustment of covariates and with FDR<0.05 as the significance level (**Figure** **3**).

### 2.4 BA features were Associated with AD Progression in a Sex‐Dependent Manner

Progression analysis was conducted on three levels. First, features associated with CN or MCI progression risk were identified by logistic regression and Cox proportional hazards regression, in men and women respectively. There were much more features associated with CN progression risk in men (n = 16) than in women (n = 2) and the number of features associated with MCI progression risk were comparable in men and women (**Table** **2**). This was in line with previous findings that BA profiles changed earlier in men than women. Out of the 34 features associated with progression risk, 10 key ones (6 for men and 4 for women; in bold in Table 2) were consistently identified by both methods and regardless of whether the covariates were adjusted or not. Among them, CA, CA%, and CDCA% were the top 3 prominent features associated with the progression risk from MCI to AD in men, and GHDCA, GHDCA%, and GHDCA/HDCA were the top 3 prominent features associated with the progression risk from MCI to AD in women.

**Table 2:** Sex‐specific feature panels associated with CN or MCI progression risk.

BA feature	logistic regression				Cox regression			
	before adjustment		after adjustment^a)^		before adjustment		after adjustment^a)^	
	p	OR(CI)	p	OR(CI)	p	OR(CI)	p	OR(CI)
CN to MCI (men‐specific panel)								
alloLCA	0.043	1.399 (1.017‐1.955)	0.023	1.495 (1.066‐2.138)				
isoLCA	0.011	1.651 (1.143‐2.484)	0.011	1.68 (1.148‐2.563)				
GHDCA	0.019	1.556 (1.095‐2.303)			0.002	1.55 (1.18‐2.04)	0.022	1.41 (1.05‐1.9)
HDCA	0.004	1.67 (1.19‐2.404)	0.018	1.527 (1.091‐2.205)				
LCA	0.030	1.44 (1.043‐2.022)	0.031	1.467 (1.045‐2.1)				
GHDCA%	0.012	1.686 (1.152‐2.604)	0.030	1.602 (1.07‐2.511)	0.019	1.44 (1.06‐1.95)	0.028	1.41 (1.04‐1.92)
GLCA%	0.041	1.436 (1.028‐2.064)	0.021	1.549 (1.085‐2.285)				
HDCA%	0.016	1.506 (1.089‐2.124)	0.026	1.471 (1.057‐2.087)				
LCA%	0.037	1.436 (1.033‐2.046)	0.027	1.486 (1.059‐2.141)				
PriBA%	0.009	0.665 (0.487‐0.899)	0.028	0.703 (0.511‐0.96)				
SecBA%	0.014	1.606 (1.123‐2.395)	0.035	1.513 (1.051‐2.268)				
Pir/Sec	0.009	0.637 (0.448‐0.886)	0.026	0.676 (0.472‐0.946)				
LCA/CDCA	0.045	1.401 (1.016‐1.969)	0.019	1.521 (1.082‐2.187)				
GLCA/CDCA			0.025	1.493 (1.061‐2.149)				
GLCA/UDCA			0.036	1.438 (1.032‐2.039)				
HDCA/HCA	0.035	1.443 (1.038‐2.055)	0.023	1.483 (1.068‐2.109)	0.016	1.96 (1.13‐3.41)		
CN to MCI (women‐specific panel)								
NorCA	0.026	1.529 (1.062‐2.255)	0.027	1.58 (1.066‐2.402)	0.014	1.48 (1.08‐2.03)		
TUDCA/UDCA	0.011	0.614 (0.414‐0.886)	0.026	0.645 (0.433‐0.938)	0.037	0.633 (0.467‐0.859)	0.027	0.654 (0.479‐0.893)
MCI to AD (men‐specific panel)								
CA	0.012	0.779 (0.639‐0.944)	0.015	0.766 (0.616‐0.946)	0.002	0.628 (0.47‐0.838)	0.001	0.603 (0.441‐0.824)
CDCA	0.032	0.81 (0.668‐0.981)	0.032	0.795 (0.644‐0.98)				
CA%	0.037	0.815 (0.672‐0.986)	0.034	0.795 (0.642‐0.981)	0.004	0.786 (0.667‐0.927)	0.002	0.758 (0.635‐0.905)
CDCA%	0.018	0.794 (0.655‐0.96)	0.014	0.769 (0.623‐0.946)	0.022	0.83 (0.707‐0.973)	0.026	0.823 (0.694‐0.976)
TCA/CDCA	0.021	1.255 (1.037‐1.525)	0.043	1.244 (1.009‐1.54)	0.038	1.17 (1.01‐1.35)	0.011	1.23 (1.05‐1.43)
TCA/CA	0.025	1.247 (1.03‐1.516)	0.049	1.239 (1.002‐1.537)	0.043	1.14 (1‐1.3)		
GCDCA/CDCA	0.038	1.227 (1.013‐1.491)	0.049	1.236 (1.003‐1.529)	0.036	1.14 (1.01‐1.3)	0.016	1.18 (1.03‐1.35)
MCI to AD (women‐specific panel)								
GCA	0.029	1.3 (1.028‐1.65)						
GCDCA	0.036	1.292 (1.019‐1.649)	0.033	1.355 (1.029‐1.799)				
GHDCA	0.002	1.511 (1.17‐1.991)	0.032	1.433 (1.042‐2.016)	0.002	1.37 (1.12‐1.68)	0.006	1.35 (1.09‐1.68)
GCDCA%	0.010	1.401 (1.093‐1.824)	0.022	1.406 (1.058‐1.9)	0.006	1.33 (1.08‐1.63)	0.044	1.26 (1.01‐1.57)
HDCA%			0.049	0.765 (0.583‐0.996)				
CA/CDCA	0.044	0.786 (0.62‐0.993)						
GCA/TCA			0.045	1.332 (1.01‐1.775)				
GUDCA/TUDCA	0.005	1.427 (1.119‐1.842)	0.043	1.34 (1.011‐1.785)			0.037	1.07 (1‐1.15)
GHDCA/HDCA	0.003	1.499 (1.159‐1.973)	0.005	1.606 (1.169‐2.264)	0.030	1.27 (1.02‐1.57)	0.015	1.3 (1.05‐1.62)

^a)^ Covariates including age, APOE‐4, education year, BMI, medications, cohort, and fasting status were adjusted. alloLCA = Allolithocholic acid, isoLCA = Isolithocholic acid, GHDCA = Glycohyodeoxycholic acid, HDCA = Hyodeoxycholic acid, LCA = Lithocholic acid, GHDCA% = the percentage of GHDCA to total bile acids, GLCA% = the percentage of GLCA to total bile acids, HDCA% = the percentage of HDCA to total bile acids, LCA% = the percentage of LCA to total bile acids, PriBA% = the percentage of all primary bile acids to total bile acids, SecBA% = the percentage of all secondary bile acids to total bile acids, Pir/Sec = the ratio of primary and secondary bile acids, LCA/CDCA = the ratio of LCA and CDA, GLCA/CDCA = the ratio of GLCA and CDCA, GLCA/UDCA = the ratio of GLCA and UDCA, HDCA/HCA = the ratio of HDCA and HDA, NorCA = Norcholic acid, TUDCA/UDCA = the ratio of TUDCA and UDCA, CA = Cholic acid, CDCA = Chenodeoxycholic acid, CA% = the percentage of CA to total bile acids, CDCA% = the percentage of CDCA to total bile acids, TCA/CDCA = the ratio of TCA and CDCA, TCA/CA = the ratio of TCA and CA, GCDCA/CDCA = the ratio of GCDCA and CDCA, GCA = Glycocholic acid, GCDCA = Chenodeoxycholic acid glycine conjugate, GCDCA% = the percentage of GCDCA to total bile acids, CA/CDCA = the ratio of CA and CDCA, GCA/TCA = the ratio of GCA and TCA, GUDCA/TUDCA = the ratio of GUDCA and TUDCA, GHDCA/HDCA = the ratio of GHDCA and HDCA.

**Figure 4:**
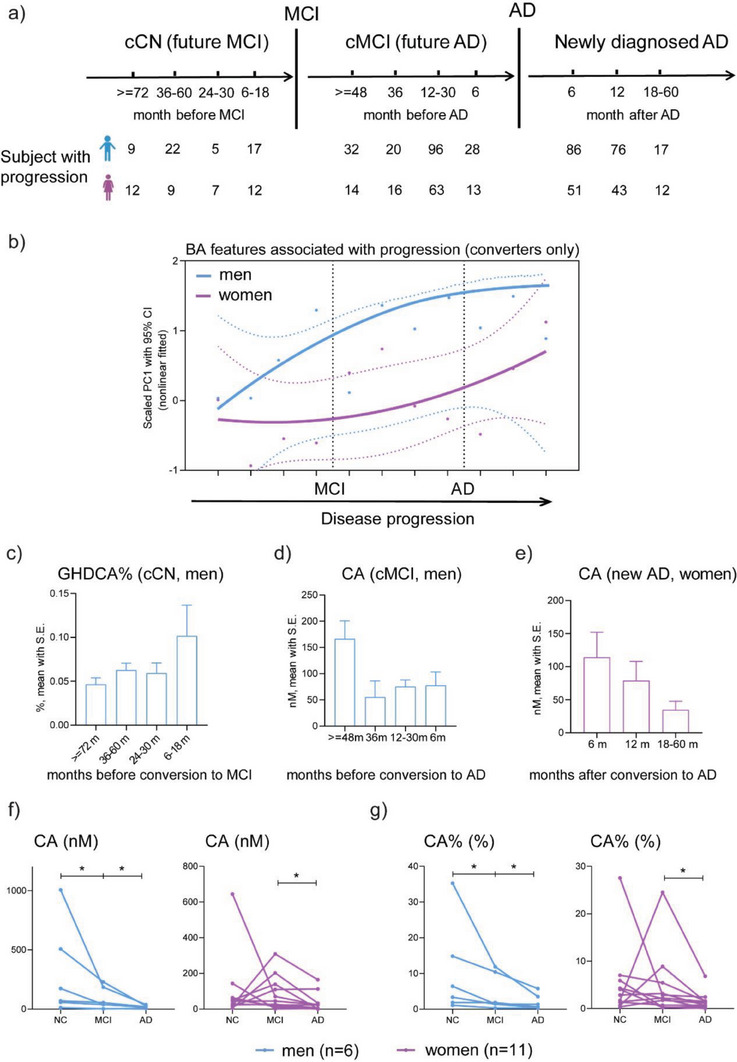
BA features were associated with AD progression in a sex‐dependent manner. a) Numbers of subjects with progression in 11 timepoints of 3 progression stages, including 4 timepoints of CNs before they converted to MCI (stage 1), 4 timepoints of MCIs before they converted to AD (stage 2), and 3 timepoints of newly diagnosed ADs after they were diagnosed as AD (stage 3). b) PCA trajectories of features associated with progression in men and women. The p values from two‐way ANOVA for timepoint, sex, and their interaction was 0.30, 0.20, and 0.56 respectively. Levels (mean with S.E.) of GHDCA% in 4 timepoints of stage 1 in men and c), CA in 4 timepoints of stage 2 in men d), and CA in 3 timepoints of stage 3 in women e). Levels (mean with S.E.) of CA f) and CA% g) in CN, MCI, and AD in subjects (6 men and 11 women) who underwent three clinical stages. * represents Wilcoxon paired singed rank test p<0.05.

Given the frequent and long‐term follow up, we examined the association between change in BA features and AD progression (three progression stages with 11 timepoints, **Figure** **4**a) in participants with stage progression (n = 660). There were 9, 15, and 1 men‐specific features and 4, 4, and 9 women‐specific features associated with each of the three stages respectively(mixed linear models with p<0.1). PCA trajectories based on these 42 features were generated. Not surprisingly, change was early and apparent in men before the diagnosis of AD. Close to and after the diagnosis of AD, change in women was increasing (Figure 4b). Levels of representative features for each of the 3 progression stages in corresponding timepoints are shown as Figure 4c–e.

Furthermore, we examined the levels of consistently significant features (p<0.05 in all the above progression analyses) in 17 subjects (men = 6, women = 11) who underwent 3 clinical stages. We found that CA and CA% decreased continuously in CN, MCI and AD in men, while in women, their levels started to decrease later, until the MCI stage (Figure 4f,g). This finding strengthens the observation that there is a delay in the changes of BA features in women compared with men.

### 2.5 BA Features Improved the Performances of Clinical Markers for Diagnosis and Progression Prediction

**Figure 5:**
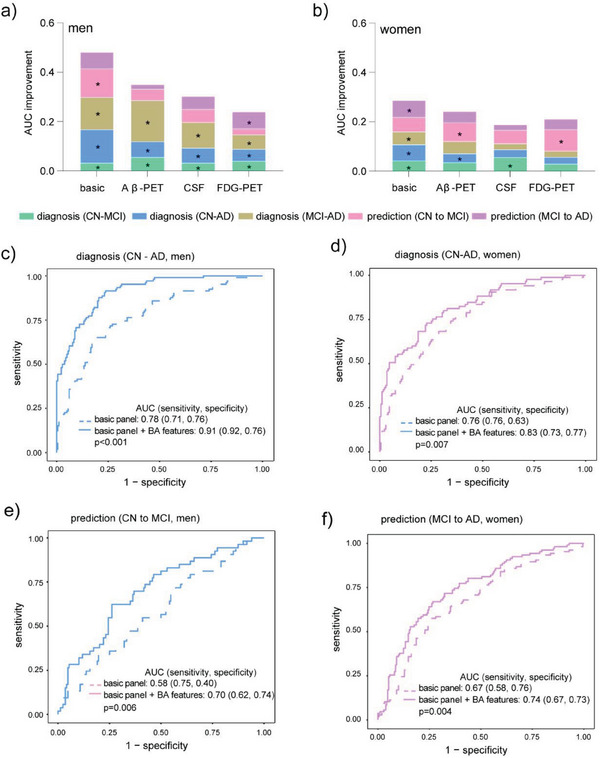
BA features improved the performances of clinical markers for diagnosis and progression prediction. Improvements in the AUC values of logistic regression models for men a) and women b), using each type of clinical marker panels following the addition of BA features for discriminating CN‐MCI, CN‐AD, and MCI‐AD and to predict future MCI and AD. The basic marker panel includes age, BMI, education year, and APOE‐4. * indicates Delong test p<0.05 comparing ROCs before and after the inclusion of BA features. ROC curves of the CN‐AD diagnostic model for men c), CN‐AD diagnostic model for women d), MCI predictive model for men e), and AD predictive model for women f), using basic markers (dash line) and basic markers combined with BA features (solid line). The p value is from Delong test.

Motivated by above findings, we assessed the contributions of BA features to clinical marker panels for AD diagnosis and progression prediction in men and women respectively. The area under receiver operating characteristic curve (AUC) values of logistic regression models based on each type of clinical marker panels and on their combination with selected BA features to discriminate between CN‐MCI, CN‐AD, and MCI‐AD and to predict future MCI or future AD are shown in **Figure** **5**a,b. All models had increased AUC values after the addition of BA features indicating the positive contributions of BA features to these panels. Comparatively, they contributed more to men (mean with S.D. 0.07 ± 0.04) than to women (0.05 ± 0.02) and made greater contributions to basic marker panel (age, BMI, education year, and *APOE‐4*) than the others. Specifically, the AUC values of models based on basic markers and BA features achieved 0.91 (men, Figure 5c) and 0.83 (women, Figure 5d) for the discrimination of CN and AD, and 0.70 (men, Figure 5e) and 0.74 (women, Figure 5f) for future MCI/AD prediction.

### 2.6 Results Validation by Refined Grouping in Subjects with CSF A and T Measurements

**Figure 6:**
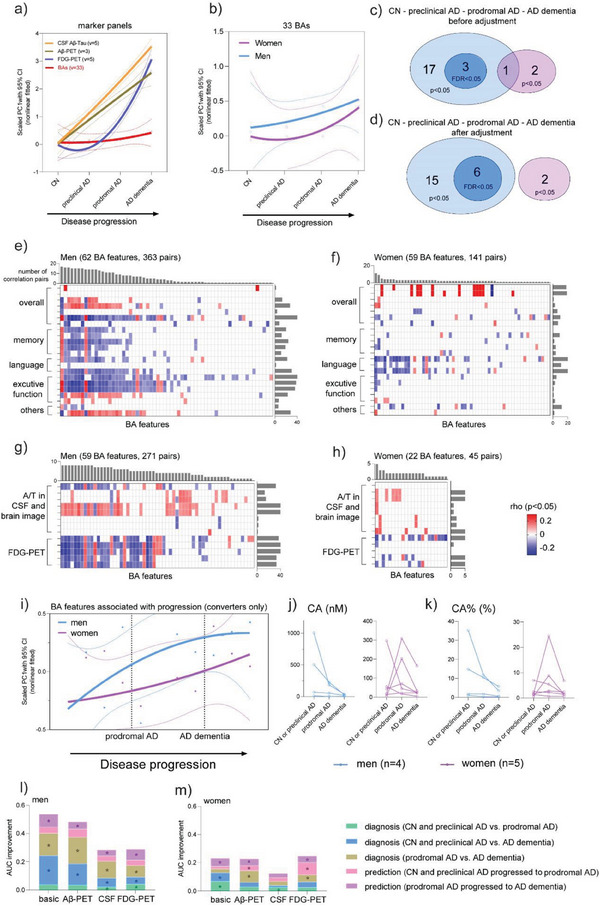
Results validation by refined grouping in subjects with C A and T. a) PCA trajectories of 6 marker panels a) and 33 BAs b) across four stages. The number of features associated with stages in men (blue) and women (purple), which were identified by mixed linear models without c) and with d) covariates adjustment. The covariates were age at sampling, *APOE‐4*, education year, BMI, medications, cohort, and fasting status. Heatmaps of the correlation coefficients (aforementioned covariates were adjusted) between 91 BA features and clinical markers from neuropsychological tests (e for men and f for women) and CSF and PET imaging (g for men and h for women). Blank, non‐significant (p>0.05); pink, positive association; blue, negative association. i) PCA trajectories of features associated with progression in men and women (converters only). Levels of CA j) and CA% (k) in subjects (4 men and 5 women) who underwent 3 stages. Improvements in the AUC values of logistic regression models for men l) and women m), using each type of clinical marker panels following the addition of BA features for diagnosis and progression prediction. * indicates Delong test p<0.05 comparing ROCs before and after the inclusion of BA features.

To better represent AD progression stages, 578 out of the 1180 subjects with both clinical diagnosis and CSF A and T tests were selected and categorized as CN (n = 61, 31 men, and 30 women), preclinical AD (n = 100, 49 men, and 51 women), prodromal AD (n = 308, 183 men, and 125 women), and AD dementia (n = 109, 62 men, and 47 women) (Table S5, Supporting Information). Once again, changes of BAs were not as dramatic as CSF and imaging markers (**Figure** **6**a), and were different in men and women (Figure 6b). More features associated with disease stages (Figure 6c,d) and clinical markers (Figure 6e–h) were identified in men than women.

During 6–120 months follow‐up, 46 subjects categorized as CN or preclinical AD progressed to prodromal AD, 172 subjects with prodromal AD progressed to AD dementia, and 175 subjects were newly diagnosed as AD dementia (Table S6 and S7, Supporting Information). Most (86% for men and 89% for women) of the features associated with the progression of MCI to AD were validated. Nine out of the 10 key features (highlighted in bold in Table 2) were still associated with progressions (Table S8, Supporting Information). Once again, the progression trajectories based on converters (Figure 6i and Figure S1, Supporting Information) and the changes of CA and CA% in 9 subjects (men = 4, women = 5) who underwent three progression stages (Figure 6j,k) suggest that there is a delay in the changes of BA features in women compared with men.

The AUC improvements of diagnostic and predictive models with and without BA features demonstrated that BA features had positive contributions to all clinical panels, with larger contributions to men (mean with S.D. 0.08 ± 0.05) than to women (0.04 ± 0.02) (Figure 6l,m).

## 3 Discussion

There is growing evidence that neurodegenerative diseases, such as AD, are closely linked to changes in the serum metabolome. Our previous study is one of the first to examine the relationship between BAs and AD development.^[^ ^5^, ^6^ ^]^ With the first longitudinal metabolome data set of ADNI, findings of this report highlight the sexually dimorphic roles of BAs in AD development and suggest their potential for clinical use.

It is well‐recognized that different types of markers have different alteration patterns and paces in the evolution of AD.^[^ ^9^ ^]^ This study characterized BA trajectories across AD progression using different grouping methods and sample sets and different variable sets (Figure 2b,c,f, and Figure 4b). These may offer novel insights into the roles of BAs in different populations, and may add new evidences for sex and stage specific diagnosis and treatment of AD.

Sex differences were extensively explored in BA profiles during AD development. More features were associated with disease stages (Figure 2d,e, and Figure 6c,d) and clinical markers (Figure 3 and Figure 6e–h) in men than women. BA abnormalities in men may precede changes in women among stage converters (Figure 2f,4b and Figure 6b,i). The differences in BA profiles and changes between sexes may be mediated by sex hormones because BAs and sex hormones share the same precursor, cholesterol.^[^ ^10^ ^]^ In agreement with our findings, BA profiles have been shown to fluctuate with the initiation and progression of sexually dimorphic diseases, such as prostate cancer,^[^ ^11^ ^]^ ovarian cancer,^[^ ^12^ ^]^ and vascular dementia.^[^ ^6^ ^]^ Meanwhile, the sexual difference may due to different expressions of BA synthetic enzymes and transporters, and gut microbiota composition.^[^ ^10^, ^13^ ^]^ It is reported in animal studies that mice treated with estrogen or androgen have been shown to exhibit significant alterations in BA profiles.^[^ ^14^ ^]^ Importantly, BAs have been used for the treatment of sex‐specific diseases.^[^ ^11^, ^15^ ^]^ We previously reported the protective effect of bile acid sequestrants against vascular dementia, with better efficacy in men than women.^[^ ^6^ ^]^ Further studies are required to elucidate the mechanisms underlying sex differences in BA profiles and their effects on AD, especially the relatively weak and/or delayed changes in women, which may pave the way for individualized medicine.

Previous research has suggested that disruptions in cholesterol homeostasis may increase the risk of developing AD.^[^ ^16^ ^]^ We have proposed that changes in both serum and brain BA profiles may be involved in the development of AD and could be potential targets for the prevention and treatment of AD.^[^ ^7^ ^]^ The results of this study support these hypotheses. The primary catabolic fate of brain and liver cholesterol is the conversion to primary BAs, CA, and CDCA, through intermediate oxysterols (e.g., 24S‐hydroxylcholesterol and 27‐hydroxycholesterol in the brain and 25‐hydroxycholesterol and 27‐hydroxycholesterol in the liver) that can cross the blood‐brain barrier.^[^ ^5^, ^7^ ^]^ Our data showed that serum levels of CA, CA%, CDCA%, and priBA% were negatively associated with disease severity (Figure 2h,j and Figure 4f,g) in men. We also observed downward trends in brain CA% from the ROS/MAP cohort (Figure S2, Supporting Information, it declined earlier in men than women). In addition, we previously observed that patients taking BA sequestrants (drugs that reduce circulating BAs and increase cholesterol catabolism) had a lower incidence of all‐cause dementia compared to patients taking other lipid‐modifying therapies, using data from the UK Clinical Practice Research Datalink (CPRD) database.^[^ ^6^ ^]^ Taken together, these findings suggest that the declines in serum and brain levels of primary BAs, CA especially, may be due to abnormalities in cholesterol catabolism, and impaired primary BA synthesis may be an important factor in AD pathology.

Noninvasive and cost‐efficient markers are desirable for AD management. Our data demonstrated that models based on basic markers and BA features had comparable performance to that of CSF and PET marker panels in men. BA features can serve as noninvasive and cost‐efficient markers for frequent and long‐term clinical monitoring. We noticed that their contributions to CSF and PET markers were not as large as those of basic markers. This may be attributable to the small sample size of CSF and PET tests and the already strong power of the CSF and PET panels. Nonetheless, BA features hold the potential for clinical use, showing remarkable improvements in combination with basic markers and positive synergistic effects with CSF and PET markers.

This study has several limitations. First, serum and brain cholesterol levels are unavailable, which are known to be associated with BA profiles and AD pathology. Second, the different change patterns and feature panels for men and women need to be validated in larger, longitudinal studies with diverse ethnic and lifestyle patterns. Third, the assessment of clinical potentials of identified features may be biased due to the small number of individuals with CSF and PET measurements. Nonetheless, our preliminary observations on the changes in BA profiles in a sex‐stratified manner offer novel insights that may help guide future pathological and clinical studies on AD.

## 4 Conclusion

In conclusion, our study highlights the significant sex differences and dynamic changes in BA profiles during AD initiation and progression. BA features have potential clinical applications, with complementing effects to certain clinical markers. Further research is needed to fully understand the gut microbiome‐BA‐brain cholesterol axis and identify potential targets for the prevention and treatment of AD.

## 5 Experimental Section

### Study Cohorts and Sample Collection

Baseline (n = 1180) and follow‐up serum samples (total n = 4219) and related information were obtained from the ADNI study (Table 1).^[^ ^17^ ^]^ Written informed consent was obtained at the time of enrollment and approved by each participating sites’ institutional review board. Complete information on ADNI study including inclusion and exclusion criteria, sample collection protocol, clinical diagnostic method, and clinical marker extraction (demographics, *apolipoprotein E* ε4 genotype (*APOE‐4*), neuropsychological test scores, cerebrospinal fluid (CSF) and positron emission tomography (PET) imaging markers) can be found at http://adni.loni.usc.edu/data‐samples/access‐data/.

### Quantitative Measurement and Pretreatment of Bile Acid Features

A total of 33 BAs were quantitatively measured and pre‐processed using an ultra‐performance liquid chromatography coupled to tandem mass spectrometry system (UPLC‐MS/MS)^[^ ^18^ ^]^ and the TMBQ software (V1.0, HMI, Shenzhen, China). Outliers were identified using Cauchy distribution robust fit (K sigma = 7). Outliers (<0.2%) and zero values (<0.1%) were replaced using multivariate normal imputation. Fifty‐eight extended BA features (Table S4, Supporting Information) were generated including percent concentrations of each BA to TBA and BA ratios reflective of enzymatic activities and gut microbiota functions. All the features were log‐transformed for statistical analysis as most of them were not normal (Shapiro‐Wilk test p<0.05).

### Clinical Marker Panels

Thirty‐nine clinical and pathological markers, categorized into six types, were included in the subsequent analysis, namely, a cognition score panel with 22 neuropsychological test scores, a CSF panel with five A and T markers derived from CSF, an Aβ‐PET panel comprising with three PET imaging markers indicating brain amyloid‐beta deposition, an FDG‐PET panel comprising five 18F‐fluorodeoxyglucose PET imaging markers associated with brain D‐glucose metabolism, a demographic panel comprising age, BMI, and education year, and an *APOE‐4* genotype status marker.

### Statistical Analyses

Differences in clinical markers were evaluated using Chi‐squared test, student's t‐test, Mann‐Whitney test, Analysis of variance, or Kruskal–Wallis test followed by Dunn's multiple‐comparison post‐hoc, as appropriate and as denoted in text and figure legends. PCA was used for dimension reduction and the first principal components (PC1s) were taken as the representative variables for corresponding marker/feature panels. Linear regression was used to correct each of the PC1s by age (sex was also corrected for Figure 2a and 6a) and scaled them by subtracting the mean and dividing by the standard deviation of the reference group (CN or the first timepoint of disease progression). Locally weighted regression (LOESS) with 95% confidence interval was used for curve fitting of PC1s (the Nonlinear regression function in Graphpad 9.3).

Mixed linear models were fitted, in men and women separately, to examine the associations between BA features and clinical stages (CN, EMCI, LMCI, and AD). The adjusted covariates include age at sampling, *APOE‐4*, education year, BMI, medications (binary variables indicating the medication classes taken by the subject, see Supporting Information for more descriptions), cohort (ADNI study phase of subject enrollment), and fasting status. The features were z‐score scaled to ensure comparable β coefficients (effect sizes). Two linear regression models were used to select medications for adjustment for each BA feature (Table S9, Supporting Information). Partial Pearson's correlation was used for association analysis between BA features and clinical markers after adjusting aforementioned covariates.

Association analysis between BA features and progression were carried out in 3 levels. First, all subjects were involved and logistic regression and Cox proportional hazards regression were conducted, in men and women respectively, to identify features associated with progression risks (discriminating CNs with and without progression to MCI; MCIs with and without progression to AD), after adjusting aforementioned covariates. Then, only progressed subjects were involved and the entire progression was divided into 3 stages with 11 timepoints (months before or after their conversions to MCI or AD). Mixed linear models were used to identify features associated with each of the three progression stages, adjusting aforementioned covariates. Finally, 17 subjects (men = 6, women = 11) who went through three progression stages were involved and the difference in feature levels was evaluated by Wilcoxon paired singed rank test. The significance level for these analyses was set as p<0.05 (two‐tailed), unless otherwise indicated.

The contribution of BA features to clinical practice was assessed according to the improvement of AUC values derived from logistic regression models based on each type of clinical marker panel alone or on clinical marker panel combined with BA features. BA features involved in diagnostic models were selected by mixed linear models adjusting aforementioned covariates (p<0.05 or 0.01). BA features involved in predictive models were those significant (p<0.05) in logistic regression or in Cox proportional hazards regression (Table 2). Models were evaluated in iterative leave one out way considering the limited sample numbers in some cases.

Defining AD by both clinical syndromes and biological markers, rather than clinical syndromic presentation alone, was becoming a unifying concept. To have a better representation of AD progression, a subset of subjects and samples with both clinical diagnostic stages and CSF A and T measurements were selected and categorized into 4 stages, CN (CN with A‐ and T‐), preclinical AD (CN with A+), prodromal AD (MCI due to AD, MCI with A+), and AD dementia (AD with A+). All the above analyses were replicated with the same methods and parameters, unless otherwise indicated.

All the data analyses were conducted using R (V3.5.1) and GraphPad (V9.3). All p‐values were adjusted using the Benjamini–Hochberg's false discovery rate (FDR) and the significance level was 0.05 (two‐tailed) unless otherwise indicated. Further details on quantitative measurement of bile acids, data quality control, medication adjustment, and so on were provided in the Supporting Information.

## Conflict of Interest

Dr. Kaddurah‐Daouk in an inventor on a series of patents on use of metabolomics for the diagnosis and treatment of CNS diseases and holds equity in Metabolon Inc., Chymia LLC and PsyProtix. Dr. Bruce S. Kristal is the inventor on general metabolomics‐related IP that has been licensed to Metabolon via Weill Medical College of Cornell University and has an equity interest in the company. All other authors declare that there are no conflicts of interests.

## Author Contributions

W.J. and R.K.D. were responsible for the concept and design of the study and led the team, which included all co‐authors. T.C., B.S.K., W.J., and R.K.D. drafted the manuscript. T.C., B.S.K., M.J.S., T.S, M.A., S.M.D., K.N., K.B., and M.L. conducted statistical analyses. L.W., G.X., T.C., and L.W. were responsible for sample analysis and quality control. Data management and medication term mapping were done by M.A., C.B., and A.K.P.. The manuscript was critically reviewed for important intellectual content by W.J., R.K.D., B.S.K., M.A., M.J.S., Q.G., X.Z., A.K.P., G.L., C.B., and Z.R..

## Supporting information

Supporting Information

## Data Availability

The data that support the findings of this study are openly available in AD Knowledge Portal at https://doi.org/10.7303/syn31513378.
