# Supplementary material for: Serum Bile Acids Improve Prediction of Alzheimer's Progression in a Sex‐Dependent Manner
Source: Adv Sci (Weinh). 2023 Dec 13;11(9):2306576. doi: 10.1002/advs.202306576 (PMC10916590; doi:10.1002/advs.202306576)
Supplement: Supplementary file 1 — Supporting Information [file ADVS-11-2306576-s001.pdf]

ADVANCED SCIENCE
Open Access

Supporting Information

for *Adv. Sci.*, DOI 10.1002/advs.202306576

Serum Bile Acids Improve Prediction of Alzheimer's Progression in a Sex-Dependent Manner

Tianlu Chen, Lu Wang, Guoxiang Xie, Bruce S. Kristal, Xiaojiao Zheng, Tao Sun, Matthias Arnold, Gregory Louie, Mengci Li, Lirong Wu, Siamak Mahmoudiandehkordi, Matthew J. Sniatynski, Kamil Borkowski, Qihao Guo, Junliang Kuang, Jieyi Wang, Kwangsik Nho, Zhenxing Ren, Alexandra Kueider-Paisley, Colette Blach, Rima Kaddurah-Daouk, Wei Jia* and for the Alzheimer's Disease Neuroimaging Initiative (ADNI) and the Alzheimer Disease Metabolomics Consortium (ADMC)*

Supporting Information

Serum bile acids improve prediction of Alzheimer's progression in a sex-dependent manner

Tianlu Chen, Lu Wang, Guoxiang Xie, Bruce S. Kristal, Xiaojiao Zheng, Tao Sun, Matthias Arnold, Gregory Louie, Mengci Li, Lirong Wu, Siamak Mahmoudiandehkordi, Matthew J. Sniatynski, Kamil Borkowski, Qihao Guo, Junliang Kuang, Jieyi Wang, Kwangsik Nho, Zhenxing Ren, Alexandra Kueider-Paisley, Colette Blach, Rima Kaddurah-Daouk[*]*, Wei Jia*[*]*, for the Alzheimer's Disease Neuroimaging Initiative and the Alzheimer Disease Metabolomics Consortium (ADMC)*

Contents

1. Study cohorts and sample collection 3

2. Quantitative measurement of metabolites 3

3. Quality control and pretreatment of metabolic data 4

4. Clinical marker panels 5

5. Statistical analyses 6

6. Brain BA levels of ROS/MAP cohort 8

7. SI Figures 9

8. SI Tables 11

1. Study cohorts and sample collection

Initially, a total of 4228 serum samples (1180 baseline subjects and their 0-120 months follow up) from the ADNI studies (2602 from ADNI-1 and 1626 from ADNI-GO2) were obtained for this study. ADNI, launched in 2003, is a longitudinal multi-center study designed to develop clinical, imaging, genetic, and biochemical biomarkers for the early detection and tracking of AD. Inclusion and exclusion criteria, clinical and neuroimaging protocols, and other information about ADNI can be found at www.adni-info.org. Demographic information, raw neuroimaging scan data, apolipoprotein E (*APOE-4*) ε4 genotype, neuropsychological test scores, and clinical information were downloaded from the ADNI data repository (www.loni.usc.edu/ADNI/). Written informed consent was obtained at the time of enrolment, which included permission for analysis and data sharing. The consent forms used were approved by each participating sites' institutional review board.

2. Quantitative measurement of metabolites

Chemicals: The standards of bile acids were obtained from Steraloids Inc. (Newport, RI) and TRC Chemicals (Toronto, ON, Canada), and the stable isotope- labeled standards were obtained from C/D/N Isotopes Inc. (Quebec, Canada) and Steraloids Inc. (Newport, RI). A series of calibration concentration of 2000, 400, 160, 32, 12.8, 2.5, and 1 nM with methanol/water (50/50, v/v) were prepared. Internal Standard (IS) concentrations were kept constant at all the calibration points at 100 nM for GCA-d4, TCA-d4, TCDCA-d9, UDCA-d4, CA-d4, GCDCA-d4, GDCA-d4, DCA-d4, and 200 nM for LCA-d4. The derivatization regents, 3-nitrophenylhydrazine (3-NPH) and N-(3-(dimethylamino)propyl)-N'- ethylcarbodiimide (EDC)-HCl were purchased from Sigma-Aldrich (St. Louis, MO).

Sample preparation: The bile acid-free matrix (BAFM) was used to prepare bile acid calibrators in order to minimize analytical variations and compensate matrix effects. The BAFM was obtained using the following charcoal-stripping protocol. A total of 3-g activated charcoal was added to 20 mL of plasma (Sigma-Aldrich, St. Louis, M.O.), respectively. The mixture was shaken at 25°C overnight, and the supernatant was obtained by ultra-centrifugation. The supernatant was further filtered with 0.22-μm membrane filter and evaluated for the

presence of bile acids before use. The calibrators were prepared in the blank matrix and processed as the same way as was used for the extraction of bile acids from the biological samples, such that the analytical variations can be monitored and the matrix effects are compensated. At the time of analysis, samples were thawed on ice-bath to diminish sample degradation. The sample preparation followed a published method with modifications [1,2]. Briefly, each 50 μL of serum, or standard solution was spiked with 150 μL of acetonitrile containing 9 internal standards and the extraction of bile acids was conducted at a laboratory shaker at 10°C and 1,500 rpm for 20 min. After centrifugation, the supernatant was transferred to a micro-centrifuge tube for lyophilization using a FreeZone freeze dryer system (Labconco, Kansas City, MO). The residue was reconstituted with 50 μL of mobile phase B (acetonitrile / methanol =95:5, v/v) and 50 μL of mobile phase A (water with formic acid, pH=3.25), and centrifuged at 13, 500 g and 4 °C for 20 min. The supernatant was transferred to a 96-well plate for LC-MS analysis and the injection volume was 5 μL.

Instrumentation: A Waters ACQUITY ultra performance LC system coupled with a Waters XEVO TQ-S mass spectrometer with an ESI source controlled by MassLynx 4.1 software (Waters, Milford, MA) was used for all analyses. Chromatographic separations were performed with an ACQUITY BEH C18 column (1.7 µm, 100 mm x 2.1 mm internal dimensions) (Waters, Milford, MA).

Data pretreatment and quantitation: The raw data files were processed using the TMBQ software (V1.0, HMI, Shenzhen, China) including peak integration, calibration, and quantification for each metabolite, according to the manufacturer's instructions. The identified peaks and calibration curves were manually checked to improve accuracy further. The laboratory staff were blinded to diagnosis and pathological data.

3. Quality control and pretreatment of metabolic data

Quality Control: All the samples were prepared and quantified by the same staff using the same protocol. Lab staffs were blinded to diagnosis and pathological data. In addition to the internal standards for quality control, two more types of QC samples, including test mixtures (a group of commercially available standards with a mass range across the system mass range

at 3 concentrations, low, medium, and high, within the range of the calibration curves), and pooled biological samples were used. The QC samples were kept at 10 °C during the entire analysis and were evenly inserted in the running sequence to monitor the stability of the analysis. Relative standard deviations (RSDs) of each metabolite in the test mixtures and pooled QC samples were calculated. The RSDs for lower concentration metabolites in the reference standard mixture were less than 30% and the RSDs for the higher concentration metabolites were better than 15%.

Data pretreatment: After unblinding and data release, metabolites were subjected to data preprocessing, including batch (plate) effect adjustment, outlier and missing value imputation, and feature extension. Out of the 33 BAs, 8 showed batch differences and were slightly adjusted using the median value of all the batches. Outliers were identified using Cauchy distribution robust fit (K sigma=7). Outliers (<0.2%) and missing values (<0.1%) were replaced using multivariate normal imputation. Based on the 33 BAs, 58 extended BA features (Table S1) were generated including mainly concentration percentages of each BA to total BAs (TBA) (e.g. the percentage of CA to total BAs, CA%) and ratios reflective of enzymatic activities (e.g. DCA/CA). The TBA was the summation of 18 BAs which can be detected stably with relatively high concentrations. They were: CA, TCA, GCA, CDCA, TCDCA, GCDCA, DCA, GDCA, TDCA, UDCA, TUDCA, GUDCA, LCA, GLCA, HCA, GHCA, GHDCA, and HDCA. Accordingly, a total of 91 BA features were used for subsequent analysis. All the features were logarithmic transformed (base=2) to normalize their distribution for statistical analysis. Among the initial 4228 study samples, nine study samples, 2 without diagnostic information and 7 replicated ones were removed, and 4129 samples (baseline=1180) were left for subsequent analysis.

4. Clinical marker panels

Six types of clinical markers were included in the subsequent analysis. The cognition panel comprised 22 neuropsychological test scores for overall cognitive function (e.g., ADAS-Cog-11, ADAS-Cog-13, MoCA, MMSE, and CDR) and four cognitive domains (memory, language, executive, and visual construction). The CSF panel included five A/T markers derived from

CSF (Aβ42, total tau, phosphorylated tau, total tau/Aβ42, and phosphorylated tau/Aβ42). The Aβ-PET panel comprised three markers indicating the brain amyloid-beta deposition (summary region SUVR normalized to whole cerebellum, summary region Centiloid normalized to whole cerebellum, and summary region SUVR normalized to composite ROI). The FDG-PET panel comprised five 18F-fluorodeoxyglucose PET imaging markers associated with D-glucose metabolism in brain (FDG SUVR in the posterior cingulate, left angular gyrus, left temporal lobe, right angular gyrus, and right temporal lobe, all normalized to pons). The demographic panel comprised 3 (age, BMI, and education year) or 4 (age, sex, BMI, and education year) variables. The *APOE* ε4 genotype carrier status was used independently or was combined with the demographic panel.

5. Statistical analyses

Differences in demographic, clinical, cognitive measurements, and the raw levels of BA features between/among groups were evaluated using student's t-test or Mann-Whitney test for two-group comparisons, as appropriate. Analysis of variance or Kruskal–Wallis test were used for multi-group comparisons followed by Dunn's multiple-comparison post-hoc for every two groups, as appropriate. Normality was evaluated using the Shapiro–Wilk test. The Chi-squared test was used for the comparisons of categorical variables.

Principal component analysis (PCA) was used for dimension reduction and the first principal components (PC1s) were taken as the representative variables for corresponding marker/feature panels. We used linear regression to correct each of the PC1s by age (sex was also corrected for figures 2a and 6a) and scaled them by subtracting the mean and dividing by the standard deviation of the reference group (CN or the first timepoint of disease progression). Locally weighted regression (LOESS) with 95% confidence interval was used for curve fitting of PC1s (the Nonlinear regression function in Graphpad 9.3).

Mixed linear models were fitted, in men and women respectively, to examine the association of BA features with clinical stages (CN, EMCI, LMCI, and AD). In each model, BA feature was set as the dependent variable. For pooled samples (baseline and follow up samples), fixed effects included disease stage, number of visits, age at the time of sampling,

APOE-4, education year, BMI, medications (see below for detailed descriptions), fasting status, and the interaction of disease stage and number of visits. Random effects were participant ID and cohort (ADNI study phase of subject enrollment). For independent samples (baseline samples only), fixed effects included disease stage, age at the time of sampling, sex, *APOE-4*, education year, BMI, medications, and fasting status. Random effect was cohort. Features were z-scored before being fed to the models and thus the β coefficients (effect sizes) were comparable.

As for the association analysis of BA features and progression, logistic regression and Cox proportional hazards regression were conducted, in men and women respectively, to identify features associated with CN progression (discriminating CN subjects who progressed to MCI and those who maintained CN during the 120 months follow up period) and MCI progression (discriminating MCI subjects who progressed to AD and those who maintained MCI), adjusting age at the time of sampling, sex, *APOE-4*, education year, BMI, medications, fasting status, and cohort. Then, we tried to identify progression related feature panels and to depict the entire progression trajectories of BA profiles in men and women. Here only progressed subjects were involved and the entire progression was divided into 3 progression stages with 11 timepoints (stage 1 included 4 timepoints of CNs before they converted to MCI, stage 2 included 4 timepoints of MCIs before they converted to AD, and stage 3 included 3 timepoints of newly diagnosed ADs after they were diagnosed as AD). Mixed linear models were used to identify features associated with each of the 3 progression stages (CN to MCI, MCI to AD, and AD duration), adjusting age, BMI, education years, *APOE-4*, fasting, cohort, and medication. After that, levels of features in 17 subjects (men=6, women=11) who went through 3 progression stages were examined by Wilcoxon paired singed rank test (CN vs. MCI and MCI vs. AD). The significance level was set as $p<0.05$ (two-tailed) for progression analysis), unless otherwise indicated.

The contribution of BA features to clinical markers in diagnosis and progression prediction were assessed by the improvement of area under ROCs (AUCs) derived from logistic regression models based on each type of clinical marker panel alone or clinical marker panel combined with BA features. BA features involved in diagnostic models were selected by

corresponding mixed linear models adjusting aforementioned covariates. BA features involved in predictive models were those significant ($p<0.05$) in logistic regression or in Cox proportional hazards regression (table 2). Models were evaluated in iterative leave one out way. In ADNI cohorts, medication and dietary supplements were systematically coded into 41 classes and intake of any medication within a category was coded as present or absent which resulted in 41 binary variables. Two linear regression models were used to select medication variables for adjustment for each BA feature, according to our published method (PubMed: 30337151 and 29039849). First, we regressed diagnostic group (CN-AD) on all medications and identified 24 ones associated with AD. Second, we regressed each BA feature on AD-associated medications and identified those associated with both AD and the BA feature. Medication variables were backward-input via Bayesian Information Criteria (BIC) to select an optimal combination of medications for preventing confounding while limiting model complexity. The medications selected for adjustment for each BA are listed in Table S2. AD medications (Cholinesterase Inhibitor and N-methyl-D-aspartate receptor antagonist) were intentionally excluded because these medications were taken only by AD and MCI participants but not by individuals with normal cognition and were largely coincided with diagnosis, leading to a highly significant correlation (Spearman $p<1.0E-100$) between medication status and diagnosis.

Defining AD by both clinical syndromes and biological markers, rather than clinical syndromic presentation alone, is becoming a unifying concept. A subset of subjects and samples with both clinical diagnostic grouping and CSF A and T measurements were selected and categorized into 4 groups, CN (CN with A- and T-), preclinical AD (CN with A+), prodromal AD (MCI due to AD, MCI with A+), and AD dementia (AD with A+). All the above analyses were replicated on the refined groups with the same methods and parameters, unless otherwise indicated.

All the data analyses were conducted using R (V3.5.1) and GraphPad (V9.3). All p-values were adjusted using the Benjamini–Hochberg's false discovery rate (FDR) and the significance level was 0.05 (two-tailed) unless otherwise indicated.

6. Brain BA levels of ROS/MAP cohort

The Religious Orders Study (ROS), which began in 1994, is a longitudinal clinical-pathologic cohort study of risk factors of cognitive decline and incident dementia run from the Rush Alzheimer's Disease Center that is comprised of individuals from religious communities (e.g., Catholic brothers, nuns, and priests) across the USA. The Rush Memory and Aging Project (MAP), which began in 1997 includes participants from northeastern Illinois, USA with a broader range of socioeconomic status and life experiences. Both studies were approved by an Intuitional Review Board of Rush University Medical Center. All subjects signed an informed consent, an Anatomic Gift Act, and a repository consent to allow their biospecimens and data to be used for ancillary studies. All research was performed in accordance with relevant guidelines/regulations set forth by the Rush University Medical Center. Both studies are conducted by the same team of examiners and share a large common core of data collection at the item level to allow for efficient merging of data.

A total of 110 brain samples (31 men and 78 women) including 51 NCs, 31 MCIs and 27 ADs were used in this study. Using targeted metabolomics protocols established in previous studies, brain BAs were quantified by the same UPLC-TQMS instrument (Waters XEVO TQ-S, Milford, USA) as that of serum samples descripted above. BAs and extended BA features were generated by the same software and formula as that of serum samples descripted above. We observed downward trends of CA% in the brains of male CN, MCI, and AD individuals (Figure S4). Unfortunately, there is no statistical significance among or between groups, presumably due to the small sample size.

7. SI Figures

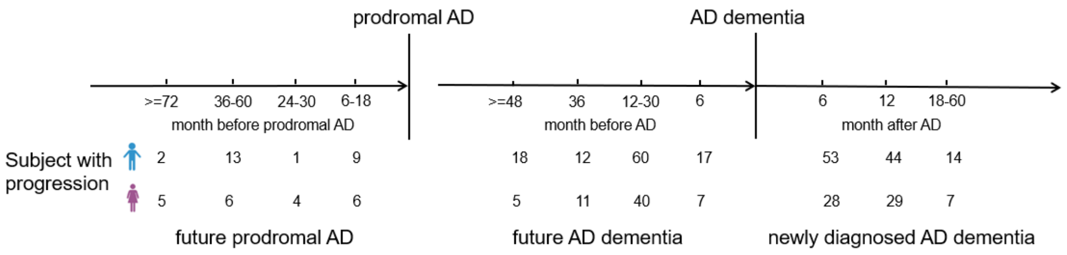

Figure S1. Numbers of subjects with progression in 3 progression stages with 11 timepoints, including 4 timepoints of subjects categorized with CN or preclinical AD before they converted to prodromal AD, 4 timepoints of subjects categorized with prodromal AD before they

converted to AD dementia, and 3 timepoints of newly diagnosed ADs after they were diagnosed as AD.

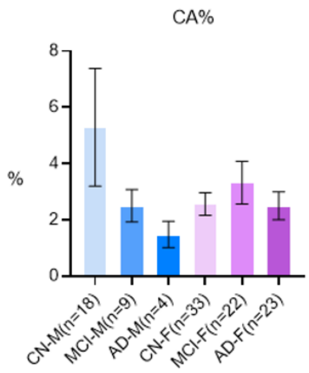

Figure S2. Levels (mean with S.E.) of CA% in CN, MCI, and AD stratified by sex in brain samples from the ROS/MAP cohort. There is no statistical significance among or between groups, presumably due to the small sample size.

8. SI Tables

Table S1. Demographic and clinical characteristics of individuals stratified by CN progression to MCI.

	sMCI(n=259)		cMCI(n=93)		p value of 2 groups	
	men	women	men	women	men	women
n	128	131	53	40		
Age(yr)	74.48±6.54	73.29±5.91	75.33±5.52	74.2±6.99	0.30	0.51
BMI	26.97±3.72	26.95±5.4	27.47±3.77	26.19±4.92	0.37	0.41
Education(yr)	17.19±2.45	15.87±2.72	16.58±2.64	15.32±2.36	0.15	0.29
APOE(e4+)%	29%	27%	25%	48%	0.57	0.01
MMSE	29.11±1.13	29.18±1.1	28.87±1.23	29.23±0.77	0.22	0.71
ADAS-11 score	6.11±2.84	4.95±2.61	7.38±2.99	6.33±3.04	0.01	0.02
ADAS-13 score	9.45±4.2	7.65±3.87	11.84±4.37	9.46±4.2	0.004	0.06

Table S2. Demographic and clinical characteristics of individuals stratified by MCI progression to AD.

	sMCI(n=355)		cMCI(n=282)		p value of 2 groups	
	men	women	men	women	men	women
n	192	163	176	106		
Age(yr)	73.57±7.5	72.02±8.14	74.76±6.55	72.86±7.67	0.06	0.35
BMI	27.42±4.05	26.89±5.42	26.35±3.57	26.05±5.84	3.09E-03	0.14
Education(yr)	16.42±2.79	15.6±2.71	16.32±2.66	15.23±2.76	0.82	0.26
APOE(e4+)%	39%	40%	62%	67%	1.53E-06	4.02E-06
MMSE	27.91±1.83	28.1±1.76	26.92±1.94	26.66±2	2.09E-07	7.39E-09
ADAS-11 score	8.99±3.98	8.21±3.88	12.78±4.3	13.11±4.83	2.06E-21	7.10E-19
ADAS-13 score	14.38±5.86	13.18±6.11	20.34±5.86	21.45±6.7	8.16E-25	1.76E-23

Table S3. Demographic and clinical characteristics of 4219 samples (collected at baseline and follow up visits from 1180 subjects) included in this study.

	All (n=4219)		CN (n=1259)		EMCI (n=691)		LMCI (n=1130)		AD (n=1139)		p value of 4 groups （NC-EMCI-LMCI-AD）	
	men	women	men	women	men	women	men	women	men	women	men	women
n	2386	1833	640	619	381	310	700	430	665	474		
Age(yr)	75.73+7.04	74.32+7.47	76.22+6.47	75.39+6.34	72.95+7.04	71.92+8.38	75.86+7.15	73.78+7.57	76.74+7.05	75.01+7.75	2.68E-16	9.12E-10
BMI	26.76+3.81	26.19+5.32	26.99+3.67	26.39+5.21	27.68+4.61	27.7+6.15	26.55+3.53	25.72+5.05	26.61+3.61	25.37+4.86	1.00E-06	3.08E-08
Education(yr)	16.45+2.76	15.29+2.78	17.03+2.51	15.67+2.72	16.59+2.61	15.44+2.64	16.22+2.89	15.43+2.78	16.03+2.84	14.57+2.82	1.94E-09	4.81E-10
APOE(e4+)%	47%	45%	26%	28%	41%	37%	51%	51%	68%	68%	<1.00E-50	1.33E-39
MMSE	26.4+3.88	26.52+4.08	28.98+1.27	29.13+1.12	28.21+1.76	28.12+1.93	27.16+2.11	26.84+2.58	22.06+4.3	21.79+4.61	<1.00E-50	<1.00E-50
ADAS-11 score	12.01+8.43	11.25+8.91	6.09+2.97	5.01+2.88	7.72+3.51	7.56+3.86	11.48+4.88	11.46+5.66	20.77+9.69	21.68+9.46	<1.00E-50	<1.00E-50
ADAS-13 score	18.51+11.17	17.32+12.43	9.6+4.41	7.77+4.21	12.42+5.29	11.89+6.1	18.57+6.83	18.56+8.28	30.81+10.92	32.4+11.11	<1.00E-50	<1.00E-50

Table S4. BA features evaluated in this study.

BA abbr	name
12-ketoLCA	12-Ketodeoxycholic acid
3-DHCA	3-Dehydrocholic acid
7-ketoLCA	7-Ketolithocholic acid
7-DHCA	7-Dehydrocholic acid
alloLCA	Allolithocholic acid
apoCA	Apocholic acid
dehydroLCA	Dehydrolithocholic acid
isoLCA	Isolithocholic acid
LCA_3S	3-Sulfolithocholic acid
muroCA	Murocholic acid
NorCA	Norcholic acid
NorDCA	Nordeoxycholic acid
TαMCA	Tauro-alpha-muricholic acid
UCA	Ursocholic acid
βUDCA	Beta-ursodeoxycholic acid
CA	Cholic acid
CDCA	Chenodeoxycholic acid
DCA	Deoxycholic acid
GCA	Glycocholic acid
GCDCA	Chenodeoxycholic acid glycine conjugate
GDCA	Deoxycholic acid glycine conjugate
GHCA	Glycohyocholic acid
GHDCA	Glycohyodeoxycholic acid
GLCA	Lithocholic acid glycine conjugate
GUDCA	Glycoursodeoxycholic acid
HCA	Hyocholic acid
HDCA	Hyodeoxycholic acid
LCA	Lithocholic acid
TCA	Taurocholic acid

TCDCA	Taurochenodesoxycholic acid
TDCA	Taurodeoxycholic acid
TUDCA	Tauroursodeoxycholic acid
UDCA	Ursodeoxycholic acid

extended BA feature	calculation formula	meaning
TBA	sum of 18 BAs; CA+CDCA+DCA+GCA+GCDCA+GDCA +GHCA+GHDCA+GLCA+GUDCA+HCA +HDCA+LCA+TCA+TCDCA+TDCA+TUDCA+UDCA	total BAs
CA%	100%*CA/TBA	percentage of CA
CDCA%	100%*CDCA/TBA	percentage of CDCA
DCA%	100%*DCA/TBA	percentage of DCA
GCA%	100%*GCA/TBA	percentage of GCA
GCDCA%	100%*GCDCA/TBA	percentage of GCDCA
GDCA%	100%*GDCA/TBA	percentage of GDCA
GHCA%	100%*GHCA/TBA	percentage of GHCA
GHDCA%	100%*GHDCA/TBA	percentage of GHDCA
GLCA%	100%*GLCA/TBA	percentage of GLCA
GUDCA%	100%*GUDCA/TBA	percentage of GUDCA
HCA%	100%*HCA/TBA	percentage of HCA
HDCA%	100%*HDCA/TBA	percentage of HDCA
LCA%	100%*LCA/TBA	percentage of LCA
TCA%	100%*TCA/TBA	percentage of TCA
TCDCA%	100%*TCDCA/TBA	percentage of TCDCA
TDCA%	100%*TDCA/TBA	percentage of TDCA
TUDCA%	100%*TUDCA/TBA	percentage of TUDCA
UDCA%	100%*UDCA/TBA	percentage of UDCA
ConBA	GCA+GCDCA+GDCA+GLCA+GUDCA+GHCA+GHDCA+TCA+TCDCA+TDCA+TUDCA	sum of conjugated BAs

UnconBA	CA+CDCA+DCA+UDCA+LCA+HCA+HDCA	sum of unconjugated BAs
PriBA	CA+CDCA+GCA+GCDCA+TCA+TCDCA	sum of primary BAs
SecBA	DCA+UDCA+LCA+HCA+HDCA+GDCA+GUDCA+GLCA+GHCA+GHDCA+TDCA+TUDCA	sum of secondary BAs
ConBA%	100%*ConBA/TBA	percentage of sum of conjugated BAs
UnconBA%	100%*UnconBA/TBA	percentage of sum of unconjugated BAs
PriBA%	100*PriBA/TBA	percentage of sum of primary BAs
SecBA%	100*SecBA/TBA	percentage of sum of secondary BAs
Pir/Sec	Pir/Sec	ratio of PriBA to SecBA
Con/Uncon	Con/Uncon	ratio of Con and Uncon
CA/CDCA	CA/CDCA	ratio of classical and alternative pathway
TCA/CDCA	TCA/CDCA	lternative pathway
GCA/CDCA	GCA/CDCA	ratio of classical and alternative pathway
GCA/TCA	GCA/TCA	glycine and taurine ratio of CA
GDCA/TDCA	GDCA/TDCA	glycine and taurine ratio of DCA
GCDCA/TCDCA	GCDCA/TCDCA	glycine and taurine ratio of CDCA
GUDCA/TUDCA	GUDCA/TUDCA	glycine and taurine ratio of UDCA
DCA/CA	DCA/CA	7a-Dehydroxylation of CA
GDCA/CA	GDCA/CA	7a-Dehydroxylation of CA
TDCA/CA	TDCA/CA	7a-Dehydroxylation of CA
LCA/CDCA	LCA/CDCA	7a-Dehydroxylation and HSDH of CDCA
GLCA/CDCA	GLCA/CDCA	7a-Dehydroxylation and HSDH of CDCA
LCA/UDCA	LCA/UDCA	7a-Dehydroxylation of UDCA
GLCA/UDCA	GLCA/UDCA	7a-Dehydroxylation of UDCA
HDCA/HCA	HDCA/HCA	7a-Dehydroxylation of HCA
UDCA/CDCA	UDCA/CDCA	HSDH of CDCA
GUDCA/CDCA	GUDCA/CDCA	HSDH of CDCA
TUDCA/CDCA	TUDCA/CDCA	HSDH of CDCA

GLCA/LCA	GLCA/LCA	glycine BSH of LCA
GHCA/HCA	GHCA/HCA	glycine BSH of HCA
GHDCA/HDCA	GHDCA/HDCA	glycine BSH of HDCA
TCA/CA	TCA/CA	taurine BSH of CA
TCDCA/CDCA	TCDCA/CDCA	taurine BSH of CDCA
TDCA/DCA	TDCA/DCA	taurine BSH of DCA
TUDCA/UDCA	TUDCA/UDCA	taurine BSH of UDCA
GCA/CA	GCA/CA	glycine BSH of CA
GCDCA/CDCA	GCDCA/CDCA	glycine BSH of CDCA
GDCA/DCA	GDCA/DCA	glycine BSH of DCA
GUDCA/UDCA	GUDCA/UDCA	glycine BSH of UDCA

Table S5. Demographic and clinical characteristics of baseline individuals stratified by refined grouping.

	All (n=578)		CN (CN A-&T-; n=61)		preclinical AD (CN A+; n=100)		prodromal AD (MCI A+ or T+; n=308)		AD dementia (AD A+ or T+; n=109)		p value of 4 groups	
	men	women	men	women	men	women	men	women	men	women	men	women
n	325	253	31	30	49	51	183	125	62	47		
Age(yr)	74.09 ± 7.0	72.52 ± 7.4	73.52 ± 5.23	70.63 ± 5.33	75.1 ± 6.81	74.33 ± 5.32	73.63 ± 7.06	71.28 ± 7.9	74.63 ± 7.68	74.63 ± 8.8	0.29	1.45E-03
BMI	26.72 ± 3.72	26.62 ± 5.53	27.63 ± 3.43	27.64 ± 4.67	26.56 ± 3.58	26.7 ± 5.35	26.9 ± 3.89	26.94 ± 6.09	25.9 ± 3.36	24.78 ± 3.83	0.13	0.08
Education(yr)	16.51 ± 2.75	15.28 ± 2.71	17.32 ± 2.31	15.1 ± 3.11	16.83 ± 2.78	15.61 ± 2.43	16.5 ± 2.73	15.39 ± 2.69	15.75 ± 2.82	14.47 ± 2.9	0.03	0.12
APOE(e4+)%	48.67%	46.53%	16.13%	13.33%	32.18%	27.59%	52.86%	55.76%	70.59%	69.39%	4.02E-08	4.48E-09
MMSE	27.19 ± 2.56	27.54 ± 2.49	29.13 ± 1.12	29.07 ± 1.01	28.93 ± 1.26	29.18 ± 0.95	27.46 ± 1.82	27.67 ± 1.89	23.16 ± 2.02	23.29 ± 1.78	8.09E-41	4.02E-32
ADAS-11 score	11.04 ± 6.04	9.42 ± 6.16	6.47 ± 3.26	4.77 ± 2.73	6.88 ± 2.96	5.28 ± 2.54	10.82 ± 4.41	9.81 ± 4.71	19.16 ± 6.6	18.31 ± 6.58	5.89E-37	3.88E-33
ADAS-13 score	17.32 ± 8.71	15.1 ± 9.46	10.02 ± 4.54	7.53 ± 4.42	10.73 ± 4.34	8.15 ± 3.7	17.26 ± 6.41	15.95 ± 7.3	29.47 ± 8.06	29.21 ± 8.11	9.66E-41	2.32E-35

Table S6. Demographic and clinical characteristics of individuals stratified by CN and preclinical AD progression to prodromal AD.

	stable CN or preclinical AD (n=115)		converted CN or preclinical AD (n=46)		p value of 2 groups	
	men	women	men	women	men	women
n	57	58	25	21		
Age(yr)	74.2 ± 7.03	72.9 ± 5.7	75.25 ± 6.05	74.62 ± 5.77	0.49	0.18
BMI	27.09 ± 3.62	26.94 ± 5.26	27.06 ± 4.27	26.86 ± 5.09	0.97	0.95
Education(yr)	17.27 ± 2.45	15.61 ± 2.65	16.0 ± 3.09	15.48 ± 2.43	0.03	0.82
APOE(e4+)%	24.76%	22.22%	38.46%	44.00%	0.16	0.03
MMSE	29.01 ± 1.25	29.17 ± 1.01	28.62 ± 1.39	28.76 ± 1.13	0.16	0.08
ADAS-11 score	6.43 ± 3.04	4.96 ± 2.47	7.72 ± 2.67	6.55 ± 4.01	0.05	0.01
ADAS-13 score	9.94 ± 4.47	7.71 ± 3.83	11.99 ± 3.54	9.95 ± 5.56	0.03	0.02

Table S7. Demographic and clinical characteristics of individuals stratified by prodromal AD progression to AD dementia.

	stable prodromal AD (n=136)		converted prodromal AD (n=172)		p value of 2 groups	
	men	women	men	women	men	women
n	72	64	107	65		
Age(yr)	73.47 ± 7.11	72.44 ± 8.2	75.09 ± 6.76	71.97 ± 7.72	0.06	0.70
BMI	27.45 ± 4.27	26.85 ± 5.69	26.12 ± 3.3	26.43 ± 6.28	0.01	0.63
Education(yr)	16.57 ± 2.79	15.61 ± 2.7	16.45 ± 2.64	15.11 ± 2.71	0.75	0.22
APOE(e4+)%	42.11%	44.29%	60.00%	73.85%	4.25E-03	7.80E-05
MMSE	27.83 ± 1.78	28.11 ± 1.79	26.91 ± 1.86	26.65 ± 2.16	6.70E-05	8.39E-07
ADAS-11 score	9.04 ± 3.73	8.19 ± 3.88	12.75 ± 4.41	13.01 ± 4.76	2.98E-12	6.19E-13
ADAS-13 score	14.51 ± 5.65	13.18 ± 6.2	20.31 ± 5.96	21.4 ± 6.27	4.27E-14	1.25E-15

Table S8. Sex-specific feature panels associated with AD progression risk (refined grouping).

BA features	logistic regression				Cox regression			
	before adjustment		after adjustment		before adjustment		after adjustment	
	p	OR(CI)	p	OR(CI)	p	OR(CI)	p	OR(CI)
**progression from CN or preclinical AD to prodromal AD (men-specific panel)**								
alloLCA								
isoLCA								
GHDCA	0.027	1.267 (1.038-1.578)			0.028	1.3 (1.03-1.64)		
HDCA	0.003	1.572 (1.168-2.143)			0.045	1.33 (1.01-1.75)		
LCA								
GHDCA%	0.049	1.211 (1.012-1.481)						
GLCA%								
HDCA%	0.035	1.274 (1.022-1.606)						
LCA%								
PriBA%								
SecBA%								
Pir/Sec								
LCA/CDCA			0.045	1.164 (1.007-1.358)				
GLCA/CDCA								
GLCA/UDCA								

HDCA/HCA					0.036	1.26 (1.02-1.57)		
**progression from CN or preclinical AD to prodromal AD (women-specific panel)**								
NorCA								
TUDCA/UDCA	0.049	0.873 (0.76-0.997)			0.005	0.845 (0.752-0.95)		
**progression from prodromal AD to AD dementia (men-specific panel)**								
CA	0.019	0.908 (0.837-0.984)	0.019	0.9 (0.823-0.982)	0.001	0.904 (0.854-0.957)	0.003	0.914 (0.861-0.97)
CDCA					0.013	0.924 (0.867-0.984)	0.030	0.929 (0.869-0.993)
CA%			0.024	0.888 (0.8-0.983)	0.003	0.908 (0.851-0.968)	0.003	0.905 (0.847-0.967)
CDCA%			0.011	0.84 (0.734-0.959)	0.035	0.915 (0.842-0.994)	0.018	0.897 (0.821-0.981)
TCA/CDCA	0.015	1.101 (1.019-1.191)	0.007	1.122 (1.032-1.222)	0.010	1.08 (1.02-1.14)	0.004	1.09 (1.03-1.16)
TCA/CA	0.014	1.089 (1.018-1.167)	0.008	1.108 (1.028-1.196)	0.001	1.08 (1.03-1.14)	0.001	1.09 (1.04-1.15)
GCDCA/CDCA								
**progression from prodromal AD to AD dementia (women-specific panel)**								
GCA					0.006	1.17 (1.04-1.3)		
GCDCA					0.003	1.2 (1.06-1.35)		
GHDCA					0.019	1.15 (1.02-1.3)		
GCDCA%					0.022	1.37 (1.05-1.8)		
HDCA%			0.020	0.777 (0.627-0.958)	0.033	0.867 (0.761-0.988)	0.044	0.864 (0.75-0.996)
CA/CDCA								
GCA/TCA					0.004	1.38 (1.11-1.71)	0.005	1.37 (1.1-1.72)

GUDCA/TUDCA	0.001	1.385 (1.152-1.687)	0.027	1.15 (1.02-1.31)
GHDCA/HDCA			0.019	1.15 (1.02-1.3)

Features were ordered as that of Table 2.

Table S9. Medications selected for adjustment for BA features.

BA feature	adjusted medication class
12-ketoLCA	PROTON PUMP INHIBITORS,VITAMIN B12 (CYANOCOBALAMIN AND ANALOGUES),FOLIC ACID AND DERIVATIVES,ASCORBIC ACID (VITAMIN C), INCL. COMBINATIONS
3-DHCA	FIBRATES
7-ketoLCA	PROTON PUMP INHIBITORS
7-DHCA	SELECTIVE SEROTONIN REUPTAKE INHIBITORS
alloLCA	PROTON PUMP INHIBITORS,NATURAL AND SEMISYNTHETIC ESTROGENS, PLAIN
apoCA	SELECTIVE SEROTONIN REUPTAKE INHIBITORS,PROTON PUMP INHIBITORS,CORTICOSTEROIDS,SOFTENERS, EMOLLIENTS
dehydroLCA	NERVOUS SYSTEM,SELENIUM
isoLCA	MINERALS
LCA_3S	SELECTIVE SEROTONIN REUPTAKE INHIBITORS,NATURAL AND SEMISYNTHETIC ESTROGENS, PLAIN
muroCA	SELECTIVE SEROTONIN REUPTAKE INHIBITORS,NERVOUS SYSTEM,NATURAL AND SEMISYNTHETIC ESTROGENS, PLAIN,FOLIC ACID AND DERIVATIVES
NorCA	DRUGS FOR URINARY FREQUENCY AND INCONTINENCE,FIBRATES
NorDCA	OTHER ANTIINFLAMMATORY AND ANTIRHEUMATIC AGENTS, NON-STEROIDS,SOFTENERS, EMOLLIENTS,FOLIC ACID AND DERIVATIVES,ASCORBIC ACID (VITAMIN C), INCL. COMBINATIONS
TaMCA	PROTON PUMP INHIBITORS,NERVOUS SYSTEM,FOLIC ACID AND DERIVATIVES
UCA	SELECTIVE SEROTONIN REUPTAKE INHIBITORS
bUDCA	SELECTIVE SEROTONIN REUPTAKE INHIBITORS
CA	SELECTIVE SEROTONIN REUPTAKE INHIBITORS,PROTON PUMP INHIBITORS,SELENIUM
CDCA	SELECTIVE SEROTONIN REUPTAKE INHIBITORS,PROTON PUMP INHIBITORS,SELENIUM

DCA	PROTON PUMP INHIBITORS,VITAMIN B12 (CYANOCOBALAMIN AND ANALOGUES),SOFTENERS, EMOLLIENTS,FOLIC ACID AND DERIVATIVES,ASCORBIC ACID (VITAMIN C), INCL. COMBINATIONS
GCA	NONE
GCDCA	BENZODIAZEPINE DERIVATIVES
GDCA	SELECTIVE SEROTONIN REUPTAKE INHIBITORS,ASCORBIC ACID (VITAMIN C), INCL. COMBINATIONS
GHCA	NONE
GHDCA	SELECTIVE SEROTONIN REUPTAKE INHIBITORS,FOLIC ACID AND DERIVATIVES
GLCA	SELECTIVE SEROTONIN REUPTAKE INHIBITORS,SELENIUM
GUDCA	BENZODIAZEPINE DERIVATIVES
HCA	NATURAL AND SEMISYNTHETIC ESTROGENS, PLAIN,MINERALS,SELENIUM,DRUGS FOR URINARY FREQUENCY AND INCONTINENCE,ASCORBIC ACID (VITAMIN C), INCL. COMBINATIONS
HDCA	SELECTIVE SEROTONIN REUPTAKE INHIBITORS,OTHER ANTIINFLAMMATORY AND ANTIRHEUMATIC AGENTS, NON-STEROIDS,NERVOUS SYSTEM,MINERALS,DRUGS FOR URINARY FREQUENCY AND INCONTINENCE
LCA	SELENIUM
TCA	MINERALS,DRUGS FOR URINARY FREQUENCY AND INCONTINENCE,H2-RECEPTOR ANTAGONISTS
TCDCA	DRUGS FOR URINARY FREQUENCY AND INCONTINENCE,OTHER ANTIEPILEPTICS
TDCA	SELECTIVE SEROTONIN REUPTAKE INHIBITORS,SELENIUM
TUDCA	NATURAL AND SEMISYNTHETIC ESTROGENS, PLAIN,OTHER ANTIEPILEPTICS
UDCA	PROTON PUMP INHIBITORS
TBA	PROTON PUMP INHIBITORS
CA%	SELECTIVE SEROTONIN REUPTAKE INHIBITORS,OTHER ANTIDEPRESSANTS,PROTON PUMP INHIBITORS,SELENIUM

CDCA%	SELECTIVE SEROTONIN REUPTAKE INHIBITORS,PROTON PUMP INHIBITORS,MINERALS,SELENIUM
DCA%	OTHER ANTIINFLAMMATORY AND ANTIRHEUMATIC AGENTS, NON-STEROIDS,VITAMIN B12 (CYANOCOBALAMIN AND ANALOGUES),ASCORBIC ACID (VITAMIN C), INCL. COMBINATIONS
GCA%	PROTON PUMP INHIBITORS,MINERALS,SELENIUM
GCDCA%	PROTON PUMP INHIBITORS,VITAMIN B12 (CYANOCOBALAMIN AND ANALOGUES),ASCORBIC ACID (VITAMIN C), INCL. COMBINATIONS
GDCA%	SELECTIVE SEROTONIN REUPTAKE INHIBITORS,OTHER ANTIDEPRESSANTS,OTHER ANTIINFLAMMATORY AND ANTIRHEUMATIC AGENTS, NON-STEROIDS,VITAMIN B12 (CYANOCOBALAMIN AND ANALOGUES),SELENIUM,FIBRATES,ASCORBIC ACID (VITAMIN C), INCL. COMBINATIONS
GHCA%	PROTON PUMP INHIBITORS
GHDCA%	SELECTIVE SEROTONIN REUPTAKE INHIBITORS,PROTON PUMP INHIBITORS,BENZODIAZEPINE RELATED DRUGS,SOFTENERS, EMOLLIENTS,TESTOSTERONE-5-ALPHA REDUCTASE INHIBITORS
GLCA%	PROTON PUMP INHIBITORS
GUDCA%	NATURAL AND SEMISYNTHETIC ESTROGENS, PLAIN,ASCORBIC ACID (VITAMIN C), INCL. COMBINATIONS
HCA%	NATURAL AND SEMISYNTHETIC ESTROGENS, PLAIN,DRUGS FOR URINARY FREQUENCY AND INCONTINENCE,FIBRATES,ASCORBIC ACID (VITAMIN C), INCL. COMBINATIONS
HDCA%	PROTON PUMP INHIBITORS,OTHER ANTIINFLAMMATORY AND ANTIRHEUMATIC AGENTS, NON-STEROIDS
LCA%	BENZODIAZEPINE RELATED DRUGS,VITAMIN B12 (CYANOCOBALAMIN AND ANALOGUES),DRUGS FOR URINARY FREQUENCY AND INCONTINENCE
TCA%	OTHER ANTIDEPRESSANTS,MINERALS,H2-RECEPTOR ANTAGONISTS
TCDCA%	OTHER ANTIDEPRESSANTS,OTHER ANTIINFLAMMATORY AND ANTIRHEUMATIC AGENTS, NON-STEROIDS,MINERALS,BULK-FORMING LAXATIVES,VITAMIN B12 (CYANOCOBALAMIN AND

	ANALOGUES),CORTICOSTEROIDS,FOLIC ACID AND DERIVATIVES,H2-RECEPTOR ANTAGONISTS
TDCA%	SELECTIVE SEROTONIN REUPTAKE INHIBITORS,SELENIUM
TUDCA%	OTHER ANTIINFLAMMATORY AND ANTIRHEUMATIC AGENTS, NON-STEROIDS,NATURAL AND SEMISYNTHETIC ESTROGENS, PLAIN,FOLIC ACID AND DERIVATIVES,H2-RECEPTOR ANTAGONISTS
UDCA%	NONE
ConBA	SELECTIVE SEROTONIN REUPTAKE INHIBITORS,SELENIUM
UnconBA	SELECTIVE SEROTONIN REUPTAKE INHIBITORS,PROTON PUMP INHIBITORS,SELENIUM,FOLIC ACID AND DERIVATIVES
PriBA	PROTON PUMP INHIBITORS
SecBA	SELECTIVE SEROTONIN REUPTAKE INHIBITORS,PROTON PUMP INHIBITORS,ASCORBIC ACID (VITAMIN C), INCL. COMBINATIONS
ConBA%	SELECTIVE SEROTONIN REUPTAKE INHIBITORS,PROTON PUMP INHIBITORS,MINERALS,SELENIUM,OTHER OPHTHALMOLOGICALS
UnconBA%	SELECTIVE SEROTONIN REUPTAKE INHIBITORS,PROTON PUMP INHIBITORS,MINERALS,SELENIUM,OTHER OPHTHALMOLOGICALS
PriBA%	SELECTIVE SEROTONIN REUPTAKE INHIBITORS,OTHER ANTIDEPRESSANTS,OTHER ANTIINFLAMMATORY AND ANTIRHEUMATIC AGENTS, NON-STEROIDS,VITAMIN B12 (CYANOCOBALAMIN AND ANALOGUES),ASCORBIC ACID (VITAMIN C), INCL. COMBINATIONS
SecBA%	SELECTIVE SEROTONIN REUPTAKE INHIBITORS,OTHER ANTIDEPRESSANTS,OTHER ANTIINFLAMMATORY AND ANTIRHEUMATIC AGENTS, NON-STEROIDS,VITAMIN B12 (CYANOCOBALAMIN AND ANALOGUES),ASCORBIC ACID (VITAMIN C), INCL. COMBINATIONS
Pir/Sec	SELECTIVE SEROTONIN REUPTAKE INHIBITORS,TESTOSTERONE-5-ALPHA REDUCTASE INHIBITORS
Con/Uncon	MINERALS,VITAMIN B12 (CYANOCOBALAMIN AND ANALOGUES),SELENIUM,FOLIC ACID AND DERIVATIVES
CA/CDCA	SELECTIVE SEROTONIN REUPTAKE INHIBITORS,MINERALS
TCA/CDCA	MINERALS

GCA/CDCA	MINERALS
GCA/TCA	H2-RECEPTOR ANTAGONISTS
GDCA/TDCA	NONE
GCDCA/TCDCA	DRUGS FOR URINARY FREQUENCY AND INCONTINENCE
GUDCA/TUDCA	NONE
DCA/CA	SELECTIVE SEROTONIN REUPTAKE INHIBITORS
GDCA/CA	SELECTIVE SEROTONIN REUPTAKE INHIBITORS
TDCA/CA	SELECTIVE SEROTONIN REUPTAKE INHIBITORS
LCA/CDCA	OTHER ANTIINFLAMMATORY AND ANTIRHEUMATIC AGENTS, NON-STEROIDS,VITAMIN B12 (CYANOCOBALAMIN AND ANALOGUES)
GLCA/CDCA	SELECTIVE SEROTONIN REUPTAKE INHIBITORS
LCA/UDCA	NONE
GLCA/UDCA	NONE
HDCA/HCA	SELECTIVE SEROTONIN REUPTAKE INHIBITORS,BENZODIAZEPINE DERIVATIVES
UDCA/CDCA	SELECTIVE SEROTONIN REUPTAKE INHIBITORS
GUDCA/CDCA	SELECTIVE SEROTONIN REUPTAKE INHIBITORS,OTHER OPHTHALMOLOGICALS,FOLIC ACID AND DERIVATIVES
TUDCA/CDCA	SELECTIVE SEROTONIN REUPTAKE INHIBITORS,MINERALS,FOLIC ACID AND DERIVATIVES
GLCA/LCA	NERVOUS SYSTEM,CORTICOSTEROIDS
GHCA/HCA	BENZODIAZEPINE DERIVATIVES
GHDCA/HDCA	NONE
TCA/CA	SELECTIVE SEROTONIN REUPTAKE INHIBITORS,PROTON PUMP INHIBITORS,OTHER OPHTHALMOLOGICALS
TCDCA/CDCA	MINERALS
TDCA/DCA	NONE
TUDCA/UDCA	FOLIC ACID AND DERIVATIVES
GCA/CA	SELECTIVE SEROTONIN REUPTAKE INHIBITORS,PROTON PUMP INHIBITORS
GCDCA/CDCA	SELECTIVE SEROTONIN REUPTAKE INHIBITORS,PROTON PUMP INHIBITORS,OTHER ANTIEPILEPTICS

GDCA/DCA	SELECTIVE SEROTONIN REUPTAKE INHIBITORS,PROTON PUMP INHIBITORS,VITAMIN B12 (CYANOCOBALAMIN AND ANALOGUES),OTHER ANTIEPILEPTICS
GUDCA/UDCA	VITAMIN B12 (CYANOCOBALAMIN AND ANALOGUES),FOLIC ACID AND DERIVATIVES
